# Remote Photoplethysmography Technology for Blood Pressure and Hemoglobin Level Assessment in the Preoperative Assessment Setting: Algorithm Development Study

**DOI:** 10.2196/60455

**Published:** 2025-06-06

**Authors:** Selene Y L Tan, Jia Xin Chai, Minwoo Choi, Umair Javaid, Brenda Pei Yi Tan, Belinda Si Ying Chow, Hairil Rizal Abdullah

**Affiliations:** 1 Division of Anesthesiology and Perioperative Medicine, Singapore General Hospital, Outram Road, Singapore, 169608, Singapore; 2 Nervotec, Singapore, Singapore; 3 Duke-NUS Medical School, Singapore, Singapore

**Keywords:** blood pressure, hemoglobin, noninvasive, telehealth, remote photoplethysmography, smartphone, hypertension, anemia, mHealth, mobile health, development, validation, hypertensive, systolic, diastolic, physiological

## Abstract

**Background:**

Blood pressure (BP) and hemoglobin concentration measurements are essential components of preoperative anesthetic evaluation. Remote photoplethysmography (rPPG) is an emerging technology that may be used to measure BP and hemoglobin concentration noninvasively with just a consumer-grade smartphone, replacing traditional in-person measurements. However, there is limited data regarding the use of this technology in patients with diverse skin tones and medical comorbidities. Hence, widespread applicability is yet to be achieved. The potential benefits of achieving this would be immense, allowing for greater convenience, accessibility, and reduction in labor and resources.

**Objective:**

Our study aims to be the first to develop an algorithm for noninvasive rPPG-based BP and hemoglobin concentration measurement that can be used for preoperative evaluation of patients in real-world clinical practice settings.

**Methods:**

We conducted the study at Singapore General Hospital from March 1, 2023, to June 28, 2024. A total of 200 patients were recruited. Our primary analysis compared the accuracy of rPPG-based systolic and diastolic BP measurements against measurements taken with automated BP measuring devices. Our secondary analysis compared the accuracy of rPPG-based hemoglobin concentration measurement against traditional blood sampling.

**Results:**

Our model performed best with diastolic BP predictions, with a mean absolute percentage error of 7.52% and a mean difference of 0.16 mm Hg (SD 3.22 mm Hg) between reference and measured readings. The 95% CI for the mean difference between predicted and measured diastolic BP was ±0.57 (−0.41 to 0.73) mm Hg. Systolic BP predictions yielded a mean absolute percentage error of 9.52% and a mean difference of 2.69 mm Hg (SD 7.86 mm Hg). The 95% CI for the mean difference between predicted and measured systolic BP was ±1.14 (−1.54 to −3.83) mm Hg. Hemoglobin concentration predictions had a mean absolute percentage error of 8.52%, with a mean difference of 0.23 g/dL (SD 0.67 g/dL). The 95% CI for the mean difference between predicted and reference measured hemoglobin concentration was ±0.10 (95% CI 0.13‐0.33) g/dL.

**Conclusions:**

Noninvasive rPPG-based measurement of BP and hemoglobin concentration at the preoperative evaluation setting has great potential for improving convenience, improving efficiency, and conserving resources for patients and health care providers. Our model was able to accurately predict diastolic BP in patients with diverse skin tones and medical comorbidities. The findings of this study serve as a basis for further studies to develop and validate the model for noninvasive rPPG-based BP and hemoglobin concentration measurement.

## Introduction

With the use of just an ubiquitous smartphone camera, remote photoplethysmography (rPPG) technology can measure vital signs remotely, eliminating the need for traditional in-person physical assessments. In telehealth and patient care applications, noninvasive vital sign measurements can transform billions of devices into cost-effective and portable health care measuring devices [1].

The major obstacles to obtaining an accurate signal for rPPG-based vital sign measurements are changes in ambient lighting conditions and movement artifacts. However, these artifacts always present and are not completely avoidable in realistic conditions [2].

Our study aims to develop a model for noninvasive, rPPG-based BP and hemoglobin concentration measurement in patients with diverse skin tones and medical comorbidities in real world conditions, at the preoperative evaluation clinic setting. Preoperative assessment is an important aspect of care for all patients undergoing anesthesia and surgery. The demand for surgical services is rising due to population growth and aging, technological advances and changes in treatment perspectives [3,4]. This in turn leads to greater demand for preoperative anesthetic assessments at the preoperative evaluation clinic. Telemedicine is an imminent adjunct that could improve patient care and the effectiveness of treatment in a rapidly aging population with an increasing number of chronic health conditions [5,6].

An essential component of preoperative assessment is the measurement of vital signs such as blood pressure (BP) and hemoglobin concentration, both of which are traditionally done in-person at the preoperative evaluation clinic setting. Preoperative hypertension is associated with increased perioperative risk [7]. Anemia is one of the strongest risk factors for perioperative blood transfusions, which could bring further complications, such as increased length of stay [8].

Automated BP measuring devices are the most commonly used devices for BP measurement [9], while hemoglobin concentration is measured with an invasive blood test. The ideal alternative is a noninvasive, contactless physiological monitor, which would provide greater comfort, convenience, and efficiency.

rPPG works on the principle that reflected light from the various regions of the skin is affected by the volume of blood under the skin [10]. This allows for an optical measurement technique that quantifies peripheral blood volume and flow variations [11]. These measurements are then analyzed with signal processing algorithms which then generate physiological measurements, such as BP [12]. Furthermore, rPPG technology relies only on a consumer-grade smartphone camera and does not require skin contact, which makes it even more ideal for use in telemonitoring and mobile health (mHealth) apps [13,14]. Developing rPPG technology for use in the preoperative evaluation clinic setting can improve manpower, save costs and lead to higher patient satisfaction scores without increasing day-of-procedure case cancellations [15,16].

An additional advantage is that rPPG technology may allow for telemonitoring of ambulatory BP, which is currently considered the gold standard for accurate diagnosis of hypertension [17]. However, the majority of patients are assessed via clinic BP measurements [18]. Home-based telemonitoring may lead to better control of ambulatory BP, especially among those with inadequate BP control [19-21].

Despite the potential advantages of contactless rPPG vital sign monitoring, there are currently some important limitations that our study aims to address. Most early studies on contactless rPPG-based BP measurement were conducted in a nonclinical setting on healthy, normotensive participants with similar skin tones [12,22,23]. These limitations must be addressed for rPPG technology to achieve widespread, commercially viable usage in the clinical setting. Our study aims to develop an rPPG model for remote BP monitoring and hemoglobin concentration estimation across a representative range of BP readings and skin tones at the preoperative evaluation clinic setting.

## Methods

### Study Design

The study was conducted at Singapore General Hospital between March 1, 2023, and June 28, 2024. Our study used a prospective feasibility design to compare contactless rPPG BP and hemoglobin concentration measurements with reference measurements from automated cuffed BP measuring devices and clinical laboratory testing. A total of 200 patients participated.

### Data Collection

**Figure 1.**
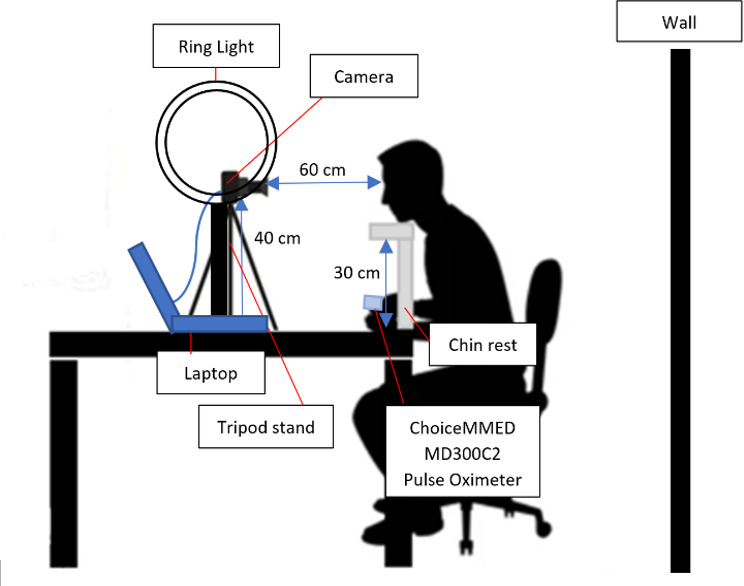
Equipment setup and patient positioning for blood pressure and hemoglobin concentration measurement with Nervotec remote photoplethysmography technology in the preoperative evaluation clinic.

The study was conducted in the preoperative evaluation clinic with Nervotec’s contactless rPPG vital signs measurement technology and equipment for standard reference measurements (automated cuffed BP measuring device and pulse oximeter), as illustrated in Figure 1. Patients were seated in a private room and positioned comfortably with their arms placed at a 90-degree angle on the table. Two individual measurements were obtained sequentially using both the Welch Allyn Connex machine and Nervotec’s rPPG technology (NervoScan). Nervotec, our technical partner, is a Singaporean digital health and artificial intelligence (AI) company that has successfully used rPPG technology in telehealth and mHealth apps to allow for the collection of rPPG waveforms via smartphones. Their base technology measures heart rate (HR), heart rate variability (HRV), respiration rate (RR) and blood oxygen saturation (SpO2) levels through signal processing techniques of a facial scan via the smartphone camera.

The NervoScan generates estimates every 10 seconds, therefore the goal was to capture the respective clinical reference for BP per 10 seconds. This was not feasible due to reasons such as limitations in the continuous noninvasive BP monitoring and waveform extraction as well as time synchronicity of the two measuring devices (NervoScan and the clinical device). To address this, the study team used a pulse oximeter in conjunction with the Welch Allyn Connex Vital Signs Monitor 6000 Series, which serves as the standard equipment within the preoperative clinic setting. The vital signs, including HR, RR, SpO2, HRV, and BP estimates, were simultaneously captured using Nervotec’s rPPG technology, recorded with a laptop and webcam setup. The parameters were collected via a synchronized measurement process lasting 3 minutes. Hemoglobin concentration was retrospectively derived from the rPPG scans.

**Figure 2.**
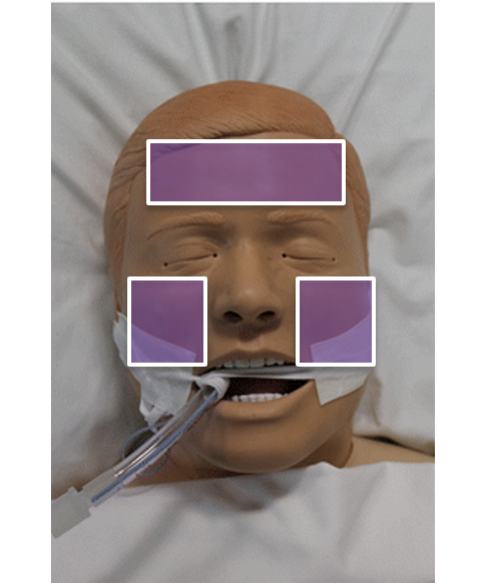
Regions of interest for the remote photoplethysmography facial scan.

Information on age, height, weight, gender, ethnicity, Monk Skin Tone (MST) Scale, relevant medical history and routine preoperative blood investigation results (full blood count, renal panel, anemia panel, coagulation, thyroid function) were collected from the patient’s electronic medical records. The MST Scale is a validated alternative to previous skin tone scales (eg, Fitzpatrick Scale), which has been adopted for multiple uses, including AI and machine learning [24]. Data collected were nonidentifiable, and only numerical data were collected. No photographic face recognition data were stored. The regions of interest captured by the rPPG facial scan were the forehead and bilateral cheeks, as indicated in Figure 2 below.

### Dataset

A total of 200 patients with a mean age of 56.44 years (range: 21‐84 y) participated. Of these patients, 67% had comorbid conditions such as hypertension, diabetes mellitus, and ischemic heart disease. The demographic profile of the participants is given in Table 1.

**Table 1.** Demographic profile of patients (N=200).

Characteristic	Patients
Sex, n (%)	
Male	76 (38)
Female	124 (62)
Age (years), mean (range)	58.23 (21-84)
SBP^a^ (mm Hg) determined from baseline reference measurement, mean (range)	127.57 (84.5‐191)
DBP^b^ (mm Hg) determined from baseline reference measurement, mean (range)	75.17 (54.5‐98)
Race, n (%)	
Chinese	160 (80)
Filipino	1 (0.5)
Malay	16 (8)
Indian	15 (7.5)
Eurasian	2 (1)
Others	6 (3)
Skin tone, n (%)	
Monk skin tone 5 or 6	118 (59)
Monk skin tone ≤4	82 (0.41)
Underlying medical conditions, n (%)	
None	66 (33)
Ischemic heart disease	6 (3)
Diabetes mellitus	48 (24)
Hypertension	80 (40)

a SBP: systolic blood pressure.

b DBP: diastolic blood pressure.

### Definitions

We defined hypertension according to the European Society of Hypertension (ESH) guidelines [25]. The ESH classifies office BP and defines hypertension grades as shown in Table 2. For our study, we combined Grade 2 and 3 hypertension into a single category. We also defined hypotension as systolic BP (SBP) ≤100 mm Hg and/or diastolic BP (DBP) ≤60 mm Hg [26].

The absolute numbers and percentages of patients in each BP category are presented in Table 3.

A more detailed breakdown of the percentages of participants in each BP category was also compared against the International Organization for Standardization (ISO) 81060-2:2018 requirements (Table 4) [27].

**Table 2.** Definition of hypertension grades.

BP^a^ classification	SBP^b^ (mm Hg) and DBP^c^ (mm Hg) criteria	
Optimal	SBP of <120 and DBP of <80	
Normal	SBP of 120‐139 and/or DBP of 80‐89	
Grade 1 hypertension	SBP of 140‐159 and/or DBP of 90‐99	
Grade 2 hypertension	SBP of 160‐179 and/or DBP of 100‐109	
Grade 3 hypertension	SBP of ≥180 or DBP of ≥110	

a BP: blood pressure.

b SBP: systolic blood pressure.

c DBP: diastolic blood pressure.

**Table 3.** Numbers and percentages of patients sampled from each blood pressure category.

BP	Hypotensive, n (%)	Grade 1 hypertension, n (%)	Grade 2 and 3 hypertension, n (%)
Systolic	16 (8)	36 (18)	16 (8)
Diastolic	8 (4)	27 (13.5)	0 (0)

**Table 4.** Percentage of patients in each blood pressure category compared with International Organization for Standardization (ISO) requirements.

BP^a^ range	Patients (%)	ISO requirements (%)
SBP^b^ ≤100 mm Hg (hypotension)	8	≥5
SBP ≥140 mm Hg (Grade 1 hypertension)	26	≥20
SBP ≥160 mm Hg (Grade 2 and 3 hypertension)	8	≥5
DBP^c^ ≤60 mm Hg (hypotension)	4	≥5
DBP ≥85 mm Hg (Grade 1 hypertension)	13.5	≥20
DBP ≥100 mm Hg (Grade 2 and 3 hypertension)	0.0	≥5

a BP: blood pressure.

b SBP: systolic blood pressure.

c DBP: diastolic blood pressure.

### Data Preprocessing

In this study, facial video recordings of 200 patients were obtained, each comprising two 3 minute sessions with concurrent ground truth BP and hemoglobin concentration measurements. For each 3-minute (180s-) video, red, green, and blue (RGB) variations of the facial regions were extracted from several predefined regions of interest (ie, forehead, left cheek, right cheek, nose and cheeks, and full face). The RGB data were segmented into 18 windows of 10 seconds each, with each window representing an independent data point. The NervoScan software was used to filter out data points where the patient’s face was not centered in the camera frame, thereby enhancing data quality and consistency. A total of 4109 data points were collected for effective training of the proposed model.

We excluded data points with extreme BP values (SBP≤80 mm Hg or ≥180 mm Hg and DBP≤60 mm Hg or ≥130 mm Hg). This addressed the class imbalance issue by removing outliers, thereby enhancing the dataset’s suitability for analysis. After filtering, 3941 data points from 197 patients remained. The shuffled dataset was then divided into training, validation, and testing sets comprising 2758, 592 and 591 data points respectively.

### Machine Learning Model and Training

Our approach used a U-Net-based model enhanced with label distribution smoothing (LDS) techniques [28]. The model optimization leveraged the Adam optimizer, chosen for its efficient handling of large datasets and adaptability in gradient-based optimization. A weighted mean squared error loss function was applied to minimize prediction error, with additional adjustments provided by a triangular LDS kernel and an inverse LDS reweighting mechanism. This configuration refined prediction accuracy, particularly in instances with label distribution imbalances.

For training, we used a batch size of 64 and conducted 300 epochs, which allowed the model to capture intricate patterns and reduce overfitting. A learning rate of 0.001 was selected to ensure a balanced convergence rate without overshooting the optimal parameters. Separate models were trained for the prediction of SBP and DBP to better capture the specific patterns associated with each measurement.

### Ethical Considerations

The study was approved by the SingHealth Centralised Institutional Review Board (CIRB Ref: 2023/2042) for the period of February 15, 2023, to December 6, 2024, and was registered on ClinicalTrials.gov (trial number: NCT06320847). All patients were recruited in the preoperative evaluation clinic by research assistants. All patients who were present in the preoperative evaluation clinic at Singapore General Hospital during the study period were screened for eligibility. The inclusion criteria were (1) all patients aged above 21 years, (2) presenting for any surgery except head and neck surgery, and (3) the ability to give informed consent. The exclusion criteria were (1) patients less than 21 years old, (2) inability to give informed consent, or (3) patients undergoing head and neck surgery. Participation was voluntary, without compensation, and written informed consent was obtained from all participants. Data collected were nonidentifiable, and only numerical data were collected. No photographic face recognition data were stored.

## Results

### Primary Analysis – SBP and DBP

The primary objective was to assess the accuracy of BP measurements from rPPG compared to automated cuffed BP measuring devices. All results are reported for a test set consisting of data points from 90 participants. The results of the primary analysis are presented in Table 5.

**Table 5.** Results of primary analysis (remote photoplethysmography–based systolic and diastolic blood pressure estimation).

Metric	SBP^a^	DBP^b^
Mean absolute percentage error (%)	9.52	7.52
Reference BP^c^ (mm Hg), mean (SD)	128.77 (19.54)	75.54 (8.39)
Predicted BP (mm Hg), mean (SD)	131.46 (11.68)	75.38 (5.16)
Difference between predicted and measured BP (mm Hg), mean difference (SD)	2.69 (7.86)	0.16 (3.22)
95% CI for mean difference between predicted and measured BP (mm Hg)	±1.14 (–1.54 to −3.83)	±0.57 (−0.41 to 0.73)

a SBP: systolic blood pressure.

b DBP: diastolic blood pressure.

c BP: blood pressure.

The results revealed a general trend of underestimation of BPs at the lower end of the range and overestimation at the higher end for both SBP and DBP. To assess the agreement and correlation between the predicted and measured values, we used a Bland-Altman plot.

These results indicate the stronger performance of the model in estimating BP using rPPG for DBP.

### Validation Against Standards

**Figure 3.**
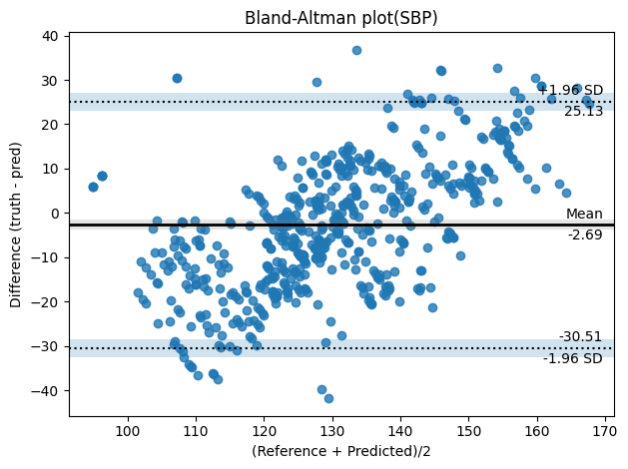
Bland-Altman plot of the differences between the reference and remote photoplethysmography–based SBP measurements. pred: predicted; SBP: systolic blood pressure.

The mean absolute differences between the predicted and measured BP measurements were 2.69 mm Hg for SBP and 0.16 mm Hg for DBP as shown in Figures3  4, respectively.

We compared the results from the NervoScan model against the ISO 81060-2:2018 standards [27]:

Using these guidelines, the performance of NervoScan BP estimates is given in Tables6  7. It can be seen that both SBP and DBP estimates satisfy the validation criterion 1 (SD≤8.0 mm Hg). Also, the DBP estimates fulfill the criterion 2 (SD<6.95 mm Hg) whereas the SBP estimates do not meet the validation criterion 2 (SD<6.30 mm Hg).

**Figure 4.**
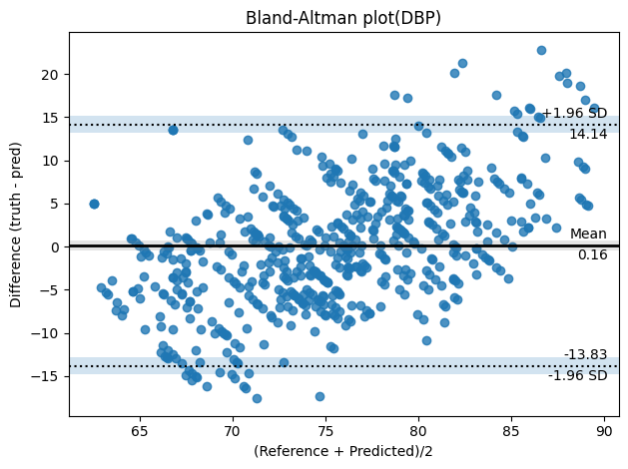
Bland-Altman plot of the differences between reference and remote photoplethysmography–based DBP measurements. pred: predicted; DBP: diastolic blood pressure.

**Table 6.** Validation results of blood pressure measurements in accordance with criterion 1 and criterion 2 of the ANSI/AAMI/ISO^a^ 81060−2:2018 guidelines (validation criteria of SD≤8.0 mm Hg for criterion 1; validation criteria of SD<6.30 mm Hg for systolic blood pressure and SD<6.95 mm Hg for diastolic blood pressure for criterion 2).

Criteria	SBP (mm Hg), estimate (SD)	Meets standard	DBP (mm Hg), estimate (SD)	Meets standard
Criterion 1	2.68 (7.86)	Yes	0.15 (3.22)	Yes
Criterion 2	2.92 (8.02)	No	0.27 (3.43)	Yes

a ANSI/AAMI/ISO: American National Standards Institute/Association for the Advancement of Medical Instrumentation/International Organization for Standardization.

**Table 7.** Validation results of blood pressure measurements in accordance with criterion 1 and criterion 2 of the guidelines.

Criteria	ISO^a^ 81060-2:2018 standard (mm Hg)	rPPG^b^ model (mm Hg)	Fulfills standards
Criterion 1 (SBP^c^)	<8	7.86	Yes
Criterion 1 (DBP^d^)	<8	3.22	Yes
Criterion 2 (SBP)	<6.3	8.02	No
Criterion 2 (DBP)	<6.95	3.43	Yes

a ISO: International Organization for Standardization.

b rPPG: remote photoplethysmography.

c SBP: systolic blood pressure.

d DBP: diastolic blood pressure.

### Statistical Analysis of SBP and DBP Predictions

To determine the appropriate statistical approach for evaluating SBP and DBP predictions by the NervoScan and respective measured readings, we performed the Shapiro-Wilk Test to check for normality. The null hypothesis states that the data follows a normal distribution. A *P* value ≤.05 indicates a statistically significant deviation from normality, leading to the rejection of the null hypothesis.

The results of the Shapiro-Wilk test are summarized in Table 8.

These findings justify the use of nonparametric statistical tests for analyzing the paired data (eg, Wilcoxon signed rank test) in subsequent comparisons.

To evaluate the agreement between predictions and ground truths for SBP and DBP, the Wilcoxon signed rank test was used, as the data consists of paired measurements. The results of the Wilcoxon signed rank test are summarized in Table 9.

In conclusion, the results highlight that while the NervoScan performs well in predicting DBP, the SBP predictions require further optimization.

**Table 8.** Shapiro-Wilk test results for systolic blood pressure (SBP) and diastolic blood pressure (DBP) measurements.

Measurement	*P* value	Interpretation
SBP	<.001	SBP does not follow a normal distribution
DBP	<.001	DBP does not follow a normal distribution

**Table 9.** Wilcoxon signed rank test results for systolic blood pressure (SBP) and diastolic blood pressure (DBP) measurements.

Measurement	*P* value	Interpretation
SBP	<.001	Significant difference between reference and rPPG^a^-based SBP measurement
DBP	.90	No significant difference between reference and rPPG-based DBP measurement

a rPPG: remote photoplethysmography.

### Secondary Analysis – Hemoglobin Concentration

**Figure 5.**
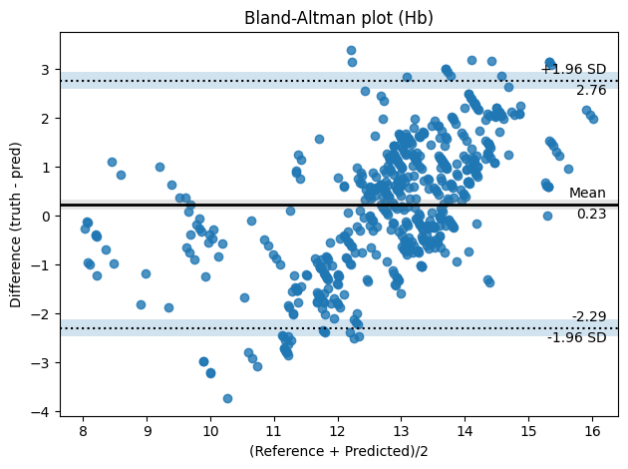
Bland-Altman plot of the difference between reference and remote photoplethysmography–based Hb concentration measurements. Hb: hemoglobin; pred: predicted.

Using the aforementioned dataset and the machine learning model architecture, a new model was trained to estimate hemoglobin. Our model predicted hemoglobin concentration with a mean absolute percentage error of 8.52%, exhibiting an error bias of 0.23 g/dL and an SD of 0.67 g/dL (Figure 5). Among 197 participants, the mean predicted hemoglobin was 12.71 g/dL (SD 1.13 g/dL), compared with the reference hemoglobin of 12.95 g/dL (SD 1.81 g/dL). The mean difference was 0.23 g/dL (SD 0.67 g/dL) as given in Table 10.

**Table 10.** Results of the secondary analysis (remote photoplethysmography–based hemoglobin concentration estimation).

Criteria	Hemoglobin (g/dL)
Mean absolute percentage error (%)	8.52
Reference hemoglobin (g/dL), mean (SD)	12.95 (1.81)
Mean predicted blood pressure (g/dL), mean (SD)	12.71 (1.13)
Difference between predicted and reference measured hemoglobin (g/dL), mean difference (SD)	0.23 (0.67)
95% CI for mean difference between predicted and reference measured hemoglobin (g/dL)	±0.10 (0.13‐0.33)

The Shapiro-Wilk test was again used to test for normality. We obtained a *P* value of <.01, which indicates that the data are not normally distributed. Furthermore, the Wilcoxon signed rank test revealed significant difference between the two hemoglobin data distributions (predictions and measured reference) with a *P* value of <.01. It is due to the inherent class-imbalance in the data distribution (majority samples are between the 12-15 g/dL range whereas fewer samples exist outside the mentioned range. Hence, the model learning/estimation is biased from such over-representation of data points from the 12‐15 g/dL range. This indicates that hemoglobin estimation can be improved by collecting sufficient hemoglobin values for both low and high values for a more effective learning.

As an initial proof of concept, this demonstrates the feasibility of hemoglobin prediction from rPPG. Thus, offering an alternative to hemoglobin estimation compared to the measurements requiring blood samples. While it offers reasonable accuracy in hemoglobin predictions compared with clinical laboratory results, the estimation can be further improved by including more data points from the low and high hemoglobin ranges. The results are demonstrated in the Bland Altman plot shown in Figure 5.

## Discussion

### Principal Findings

Our study successfully developed an rPPG model which performed strongly in predicting DBP in the preoperative evaluation clinic setting. Our model performed best with DBP predictions, with a mean absolute percentage error of 7.52% and a mean difference of 0.16 mm Hg (SD 3.22 mm Hg) between reference and measured readings, outperforming previous studies [29]. The focus of our study was to develop a contactless rPPG vital sign monitoring technology that could be used in diverse patient populations to measure BP and hemoglobin concentration in the preoperative evaluation clinic setting. Our study substantiates the potential efficacy of the rPPG-based model for accurately predicting BP and hemoglobin measurements. Further optimization is required for SBP and hemoglobin concentration predictions, as well as predictions in extreme physiological ranges. However, this development study serves as a promising basis to guide and optimize future studies, which will further develop and validate the model.

There were some limitations that we considered in our study. One limitation faced was the lack of a reference protocol for contactless vital signs monitoring. In view of this, we opted to use the American National Standards Institute (ANSI)/Association for the Advancement of Medical Instrumentation (AAMI)/ISO 81060-2:2018 standard as a guide, as automated cuffed BP measuring devices are the standard measurement device in the clinical setting. For future studies, a potential improvement would be to perform 3 reference readings per patient, instead of 2 as we have done in our study. Including more patients with extremes of BP and hemoglobin would improve the size and diversity of the dataset, which could improve model robustness. Another limitation was that the patients were not screened for arrhythmias, which would potentially affect the accuracy of contactless rPPG vital signs measurements [30]. This would also be a point of consideration for future follow-up studies.

The reported findings are promising, given that our study is the first rPPG BP and hemoglobin measurement development study based on patients with diverse skin tones, nonnormotensive BP readings and comorbid medical conditions such as diabetes mellitus and ischemic heart disease. Despite the challenges of developing a model in this heterogeneous dataset, our model was able to predict DBP accurately and achieve good correlation for SBP and hemoglobin. More work needs to be done in order to achieve the same degree of agreement for SBP and hemoglobin predictions. In doing so, we recognize the potential utility for this technology in the hospital setting, due to its wide applicability. The potential applications for this technology are vast and not just limited noninvasive BP and hemoglobin measurement in the preoperative evaluation clinic setting.

By stratifying patient readings into predefined BP and Hb categories, a successful model can significantly optimize the triaging process, thereby reducing the workload on health care professionals and increasing the efficiency of patient management. In-hospital applications would include continuous contactless monitoring in the emergency department, post-anesthesia care unit or intensive care unit This would possibly decrease human errors and improve patient safety without increasing the strain on human resources [31].

Furthermore, this model can be integrated into a mobile app (mHealth) that supports the trend toward more individualized and accessible health care solutions. Such an app could enhance ambulatory chronic disease management by enabling continuous monitoring and early detection, potentially reducing emergency visits and associated health care costs. Primary and ambulatory care patients may self-monitor their vital signs continuously and conveniently, which could be used to diagnose hypertension [32] without physically traveling to a health facility. This may allow timely intervention while reducing unwarranted hospital visits and transport costs [33]. Apart from hypertension, potential expansions may include the management of other common conditions such as diabetes [32]. For example, rPPG technology could be developed upon to measure glycated hemoglobin to diagnose diabetes mellitus and monitor glucose control in patients known to have diabetes [34].

In summary, the results of our study support further data evaluation and development of clinical applications for our rPPG model.

### Conclusions

Our study was the first development study of a model for rPPG-based, contactless, noninvasive BP and hemoglobin measurement in a population with diverse skin tones and medical comorbidities. Predictions were stronger for DBP than SBP. Predictions for SBP, DBP, and hemoglobin were most accurate in the midquartile ranges. Our study serves as a promising basis for future development and validation studies. Further studies with a larger dataset including more measurements in the extreme ends of physiological range are required. In addition, rPPG-based contactless monitoring has vast applications beyond BP and hemoglobin measurement in the preoperative evaluation clinic setting, which could potentially include other physiological measurements, such as glycated hemoglobin measurements. These broader applications are potentially limitless and could include home ambulatory monitoring, noninvasive monitoring in pediatric populations, monitoring in low-resource settings, and reducing the labor costs of monitoring patients in critical care areas in the hospital.

## Abbreviations

AAMI: Association for the Advancement of Medical Instrumentation
AI: artificial intelligence
ANSI: American National Standards Institute
BP: blood pressure
DBP: diastolic blood pressure
ESH: European Society of Hypertension
HR: heart rate
HRV: heart rate variability
ISO: International Organization for Standardization
LDS: label distribution smoothing
mHealth: mobile health
MST: Monk Skin Tone
RGB: red, green, and blue
rPPG: remote photoplethysmography
RR: respiration rate
SBP: systolic blood pressure
SpO2: blood oxygen saturation
